# Do Precision and Personalised Nutrition Interventions Improve Risk Factors in Adults with Prediabetes or Metabolic Syndrome? A Systematic Review of Randomised Controlled Trials

**DOI:** 10.3390/nu16101479

**Published:** 2024-05-14

**Authors:** Seaton Robertson, Erin D. Clarke, María Gómez-Martín, Victoria Cross, Clare E. Collins, Jordan Stanford

**Affiliations:** 1 School of Health Sciences, College of Health, Medicine and Wellbeing, The University of Newcastle, Callaghan, NSW 2308, Australiaclare.collins@newcastle.edu.au (C.E.C.); 2 Food and Nutrition Research Program, Hunter Medical Research Institute, New Lambton Heights, NSW 2305, Australia

**Keywords:** prediabetes, metabolic syndrome, personalized nutrition, precision nutrition, medical nutrition therapy, systematic review, randomized controlled trial

## Abstract

This review aimed to synthesise existing literature on the efficacy of personalised or precision nutrition (PPN) interventions, including medical nutrition therapy (MNT), in improving outcomes related to glycaemic control (HbA1c, post-prandial glucose [PPG], and fasting blood glucose), anthropometry (weight, BMI, and waist circumference [WC]), blood lipids, blood pressure (BP), and dietary intake among adults with prediabetes or metabolic syndrome (MetS). Six databases were systematically searched (Scopus, Medline, Embase, CINAHL, PsycINFO, and Cochrane) for randomised controlled trials (RCTs) published from January 2000 to 16 April 2023. The Academy of Nutrition and Dietetics Quality Criteria were used to assess the risk of bias. Seven RCTs (*n* = 873), comprising five PPN and two MNT interventions, lasting 3–24 months were included. Consistent and significant improvements favouring PPN and MNT interventions were reported across studies that examined outcomes like HbA1c, PPG, and waist circumference. Results for other measures, including fasting blood glucose, HOMA-IR, blood lipids, BP, and diet, were inconsistent. Longer, more frequent interventions yielded greater improvements, especially for HbA1c and WC. However, more research in studies with larger sample sizes and standardised PPN definitions is needed. Future studies should also investigate combining MNT with contemporary PPN factors, including genetic, epigenetic, metabolomic, and metagenomic data.

## 1. Introduction

Prediabetes is a metabolic state characterised by disruptions in glucose regulation and insulin resistance, wherein blood glucose levels exceed normal thresholds but do not reach the diagnostic criteria for Type 2 Diabetes Mellitus (T2DM) [1,2,3]. Conversely, metabolic syndrome (MetS) is a cluster of metabolic abnormalities that includes hypertension, central obesity, insulin resistance, and atherogenic dyslipidaemia [4,5]. As of 2022, the global prevalence of impaired fasting glucose is estimated at 10.6% (541 million individuals) [6], while MetS prevalence ranges from 12.5% to 31.4% [7].

The pathology for both conditions is complicated, with lifestyle, environmental, and genetic factors involved in disease progression [2,8,9]. Shared lifestyle risk factors for prediabetes and MetS include poor dietary habits, sedentary behaviour, obesity, smoking, and inadequate sleep [10]. If left untreated, prediabetes stands out as a pivotal risk factor for the eventual development of T2DM, with around 70% of individuals progressing from prediabetes to T2DM [11]. Similarly, MetS amplifies the risk not only for T2DM but also for cardiovascular disease (CVD), stroke, and myocardial infarction [2,12]. Consequently, early interventions addressing these shared risk factors are imperative to prevent adverse health outcomes associated with these conditions.

A healthy diet is widely recognised as a crucial factor in reducing the risk of prediabetes, MetS, and other non-communicable diseases [13,14]. However, current approaches to providing universal dietary recommendations or guidelines do not consider individual variations in dietary response. Personalised and precision nutrition approaches aim to improve health and well-being by leveraging dietary interventions that accommodate human variability [15]. For example, research has shown that individuals consuming the same meal may experience different glycaemic responses, highlighting the limitations of generic approaches [16]. Machine learning algorithms have also been developed to accurately predict personalised post-prandial glucose response to foods [16]. The algorithm was evaluated using a dietary intervention RCT that demonstrated a significantly lower post-prandial blood glucose response in participants after consuming lower carbohydrate, higher fibre, or higher fat-to-carbohydrate ratio meals, but this response was not consistent between individuals [16].

Currently, there is no universally agreed-upon definition for personalised and precision nutrition, and these terms are often used interchangeably. Efforts have been made to clarify these terms, with personalised nutrition defined as incorporating various information, including genetics, phenotypic, medical, nutritional, and other relevant information, to provide tailored nutritional guidance for individuals [15]. It also allows for interventions to be tailored based on an individual’s behaviour, preferences, lifestyle, and health objectives. These principles align with Medical Nutrition Therapy (MNT) [17], a category of personalised nutrition provided exclusively by registered and accredited practising dietitians. MNT involves a nutritional diagnosis and counselling services to facilitate lifestyle changes. On the other hand, precision nutrition is suggested to take a more dynamic approach, integrating genetic, metabolic, and environmental factors to develop comprehensive recommendations for individuals or subpopulation groups, utilising cutting-edge technologies such as metabolomics, metagenomics, and epigenetics [15]. In the context of this review, personalised and precision nutrition (PPN) are used as an umbrella term to encompass approaches that utilise one or more of the abovementioned components to tailor interventions to individuals. 

To date, no systematic review has summarised the evidence of MNT and PPN interventions in adults with prediabetes or MetS. Therefore, the aim of this systematic review is to consolidate current literature from randomised controlled trials investigating the effectiveness of PPN interventions, including MNT, on outcomes related to glycaemic control, anthropometry, blood lipids, blood pressure, and dietary intake among individuals with prediabetes or MetS. Findings from this review may inform future treatment and research in prediabetes or MetS through the use of PPN and/or MNT.

## 2. Materials and Methods

### 2.1. Protocol and Registration

This systematic review was conducted following the PRISMA (Preferred Reporting Items for Systematic Reviews and Meta-Analyses) guidelines (Table S1) [18]. The protocol for this systematic review was registered on Open Science Frame (OSF) (https://doi.org/10.17605/OSF.IO/9Z8TE, accessed on 20 April 2024) [19]. 

### 2.2. Database and Search

The search strategy was developed with the help of a research librarian. Medical Subject Headings and keywords were used, including terms like “prediabetes” or “risk of diabetes” and “nutrition therapy” or “personalised diet”. The search was carried out systematically across six databases (Scopus, Medline, Embase, CINAHL, PsycINFO, and Cochrane) and included articles published between January 2000 and 16 April 2023 to account for significant advancements made in PPN and MNT methods within this time and ensuring that the results of this review reflect the most up-to-date knowledge available. The search was also restricted to include only randomised controlled trials (RCTs), articles published in English, and studies involving human subjects. The complete search string for all databases can be found in Supplementary Materials, Figures S1–S6.

### 2.3. Study Selection Criteria

The inclusion of studies was determined according to the Population, Intervention, Comparison, Outcomes, and Study (PICOS) framework (as detailed in Table 1). The study population comprised adults diagnosed with prediabetes or MetS who participated in an RCT that reported the effect of a personalised nutrition-based dietary intervention, including MNT. Valid comparator groups comprised those receiving usual or standard care or engaging in non-personalised dietary interventions.

The primary outcome measures were focused on glycaemic control indicators, including HbA1c levels, fasting blood glucose concentrations, post-prandial glucose/results from oral glucose tolerance tests (OGTT), and more (Table 1). The secondary outcome measures encompassed anthropometric parameters (weight, waist circumference, and body mass index [BMI]) as well as assessments of blood lipids, blood pressure, and dietary intake.

### 2.4. Study Selection

Studies from the search results were managed using the Covidence 2.0 platform [Covidence systematic review software, Melbourne] [20]. Duplicates were removed before at least two reviewers independently screened titles, abstracts, and full texts for inclusion in this review. Discrepancies were resolved by consensus or adjudication by other research team members.

### 2.5. Risk of Bias and Study Quality Assessment

Two independent reviewers assessed the methodological quality of the included full-text articles. The risk of bias was assessed using the Academy of Nutrition and Dietetics Quality Criteria checklist [21]. This tool was selected because it has a higher inter-observer agreement than ROB 2.0 [22]. Differences were resolved by discussion and consensus.

### 2.6. Data Extraction

A standardised template, implemented within a Microsoft Excel (Version 2402; Build 16.0.17328.20282) spreadsheet, was used to extract data from the included articles. The standardised template was first piloted with three articles to ensure that all information relevant to this systematic review was collected. Data extraction was carried out by a singular reviewer (SR), and accuracy was confirmed by a second reviewer.

### 2.7. Synthesis of Results

Due to the heterogeneity in study design, population, and interventions, a meta-analysis was not performed. The results were synthesised narratively and summarised by study characteristics (study design, intervention, and control type) and outcome measure results. Wherever possible, results for outcome measures included both the confidence interval and *p*-value for within-group or between-group differences. The summary also included the consistency of the reported significant differences (*p* < 0.05) between the intervention and comparator groups for each outcome, whether the differences were increases or decreases.

## 3. Results

### 3.1. Search Results

**Figure 1:**
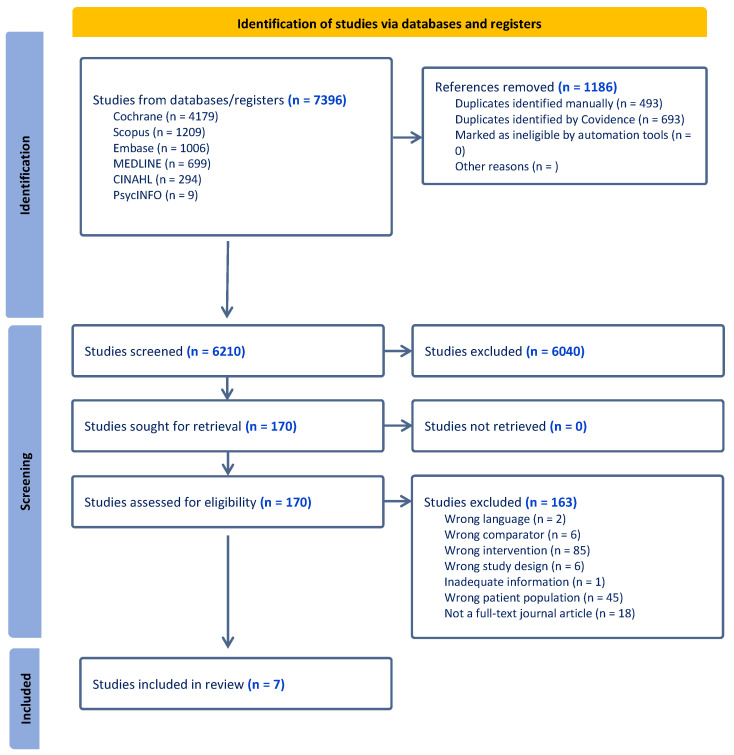
PRISMA flow diagram for the literature search and the study selection process [30].

The initial search yielded 7396 studies. After removing 1186 duplicates, 6040 articles were excluded based on their title and abstract. A total of 170 full-text articles were retrieved, and 163 studies were excluded during screening. The primary reason for exclusion was incorrect intervention type (n = 85), with most excluded interventions lacking personalisation or comprehensive lifestyle interventions involving both diet and physical activity that could not identify separate dietary impacts (Figure 1). A total of seven articles met all inclusion criteria and were included in this review [23,24,25,26,27,28,29].

### 3.2. Characteristics of the Included Studies

Characteristics of the included studies are presented in Table 2. All seven of the included papers were parallel RCTs [23,24,25,26,27,28,29]. The sample size of participants varied from 46 [26] to 225 [24], with a mean age range of 50 to 60 years. Across the included articles, there was a total of 873 participants [23,24,25,26,27,28,29]. The portion of males ranged from 28% [27] to 100% [23], and in total, across the included studies, 462 participants were male, and 411 were female [23,24,25,26,27,28,29]. Two studies were based in the USA [27,28], while others were from Brazil [29], Finland [26], Italy [25], Israel [24], and Japan [23]. Two papers met the inclusion criteria for prediabetes [24,28], two papers included individuals with MetS [25,26], two papers included those with impaired glucose tolerance [23,29], and one included participants with an HbA1c range of 6–6.9% [27].

### 3.3. Quality Assessment of Studies

The results of the quality assessment are summarised in Table S2 in Supplementary Materials. Most evaluations (n = 6) received positive ratings, with just one study rated as neutral. The neutral assessment was attributed to factors such as the absence of a power calculation and lack of blinding in the study design.

### 3.4. Interventions

Personalisation varied between studies, with four studies personalising based on dietary and nutritional information [23,25,26,27], one employed a published algorithm, which integrates clinical and gut microbiome features to predict personal post-prandial glycaemic responses to meals [24], and two were MNT interventions (Table 2) [28,29]. Four interventions were conducted by nutritionists [23,25,26,29], two by dietitians [24,28], and one by an interventionist with unreported qualifications [27]. Four interventions were delivered face-to-face [25,26,28,29], one used a combination of face-to-face and phone [27], another used a combination of face-to-face and mail [23], and one used a combination of face-to-face, email, and phone [24]. The length of the included studies ranged from 6 months [27] to 2 years [25]. The frequency of each intervention session ranged from once-off [23] to 36 sessions [29], while the duration of interventions ranged from 3 months [28] to 2 years [25]. One study compared individual MNT (considered the intervention) to group MNT (considered the comparator) [28].

### 3.5. Comparators

The comparator groups also varied among the studies (Table 2). The most common control group was a usual care comparator or standardised generic information [23,25,27]. Another study implemented a Mediterranean diet, which included specific nutrient targets based on percentage energy intake. Additionally, meals were assessed and scored based on the recommendations of four independent dietitians, and participants’ dietary preferences were also considered [24]. Among the remaining studies, one encouraged participants of the control group to continue with their usual diet and physical activity [26], and another had participants attend group nutrition sessions facilitated by a dietitian [28]. One study provided insufficient details regarding the control group, with the authors stating that participants did not receive any advice [29].

### 3.6. Outcome Measures

#### 3.6.1. Glycaemic Control Outcomes

All included studies evaluated blood glucose levels (BGLs), of which six studies reported examining fasting BGLs [23,24,26,27,28,29]. Two studies used continuous glucose monitors (CGMs) to examine mean glucose [24,27], while three examined post-prandial glucose (Table 2 and Table S3) [23,24,29].

**Figure 2:**
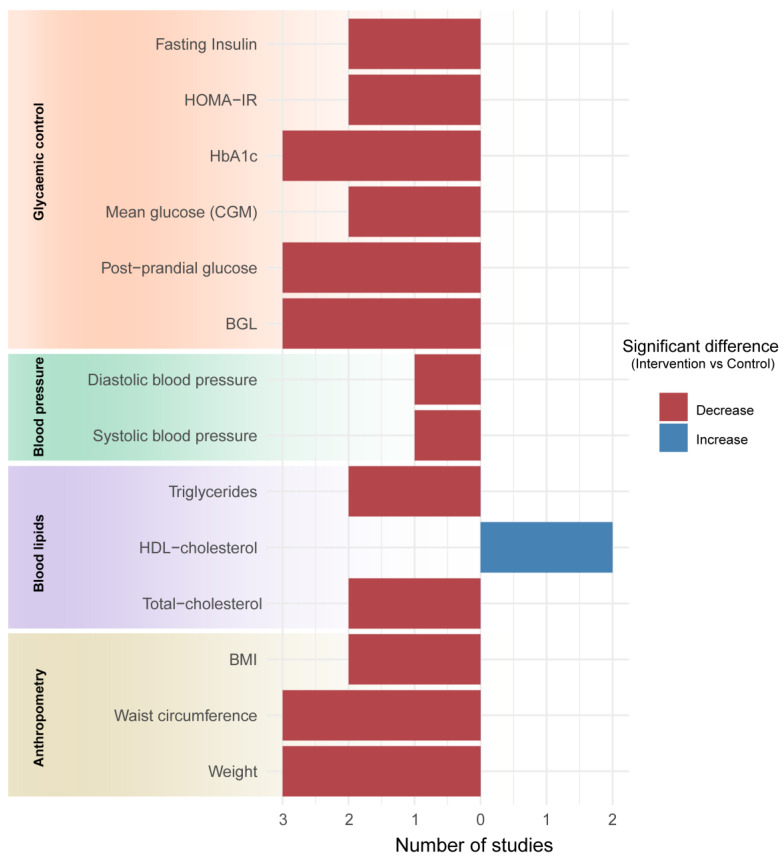
Number of studies that reported statistically significant differences between the intervention and comparison groups for each outcome of interest. The blue bars represent reported increases in the intervention group compared to the control group, while the red bars denote decreases. Studies that examined the outcome but did not report a significant between-group difference are not included in this figure (see Table S3 for further information). Esposito et al. [25] did not specify if blood/plasma glucose levels (counted under BGL outcome) were measured in a fasted state. Pimentel et al. [29] did not report the timing for when post-prandial glucose or insulin were measured, while Ben-Yacov et al. [24] calculated post-prandial glucose from continuous glucose monitor (CGM) data. BGL (Blood Glucose Level), BMI (Body Mass Index), HbA1c (Glycated Haemoglobin), HOMA-IR (Homeostatic Model Assessment of Insulin Resistance).

Four studies examining fasting BGLs reported significant decreases (*p* < 0.05) in the intervention group compared to baseline [26,27,28,29]. Two of these papers reported significant differences between the intervention and comparison groups (Figure 2) [27,29]. Another study also reported a significant difference in BGLs between the intervention and control groups but did not specify whether these measures were taken in a fasted state [25].

Both papers that measured mean glucose using a CGM reported significant differences between groups in favour of the intervention groups [24,27]. Interestingly, Dorans et al. also noted significant improvements in CGM night-time glucose in the intervention relative to the control [27].

Among the three studies that evaluated the impact on post-prandial glucose, all reported a significant difference between the intervention and control groups, although at varying times (Table 2, Figure 2, and Table S3). One study found a significant difference at 2 h after a 75 g oral glucose challenge but not at 1 h [23], while the other did not specify a time frame or dose [29]. The third study, which used CGM data, noted a difference between groups at 5 h but not 2 h following an at-home 75 g oral glucose challenge [24].

Four included studies examined HbA1c, of which three reported significant between-group differences in favour of the intervention group (Figure 2 and Table S3) [24,27,29]. Notably, in the one study that reported no difference, the intervention involved only two sessions with participants [28] compared to interventions with 8 [24], 10 [27], and 36 [29] sessions in the other studies. Ben-Yacovet et al. [24] was the only study that measured fructosamine (a shorter-term measure reflecting 2–3 week changes in blood glucose) and found a significant decrease in the intervention compared to the control group. 

Multiple papers reported outcomes related to insulin, including HOMA-IR and fasting insulin (Table 2 and Table S3) [24,25,26,27,29]. Four studies examined HOMA-IR using the homeostasis model of assessment [24,25,27,29], of which two reported a significantly greater decrease in the intervention group compared to the control group (Figure 2) [25,27]. Similarly, two of the five studies examining fasting insulin levels [24,25,26,27,29] also reported a significant between-group difference in favour of the intervention [25,27]. Pimentel et al. [29] also reported a significant decrease in post-prandial insulin levels in the intervention compared to the control group but did not indicate the timing of these measurements.

#### 3.6.2. Anthropometric Outcomes

Six of the included studies reported anthropometric data (Table 2) [24,25,26,27,28,29]. All six studies examined body weight [24,25,26,27,28,29]. Three of these studies reported significant between-group differences in favour of the intervention group (Figure 2) [25,26,27]. Four studies examined waist circumference changes [24,25,26,27], three of which reported significant differences between the intervention and control groups [25,26,27]. Five studies examined changes in BMI [24,25,26,28,29], two of which reported a significant decrease in the intervention group and a significant difference between the intervention and control groups [25,26]. 

#### 3.6.3. Blood Lipids

Five studies examined changes in blood lipid levels (Table 2) [24,25,27,28,29]. All five studies reported changes in total cholesterol [24,25,27,28,29]. Of those five, two reported significant differences between the intervention and control groups (Figure 2) [25,29]. All five studies reported changes in HDL cholesterol [24,25,27,28,29]. Of those five studies, two reported a significantly greater increase in HDL in the intervention group compared to the control group [24,25]. Four papers examined LDL cholesterol, and all papers reported non-significant between-group differences [24,27,28,29]. Four papers reported changes in triglyceride levels [24,25,28], and two reported a significant decrease in the intervention compared to control groups [24,25]. 

#### 3.6.4. Blood Pressure

Five of the included studies examined changes in systolic and diastolic blood pressure (Table 2) [24,25,26,27,28]. Only one study reported significant differences in systolic and diastolic blood pressure between the intervention and control groups, with the intervention group showing a significant decrease (Figure 2) [25]. Another study reported a significant difference between the intervention and control groups at the midway assessment (3 months) but not at the final follow-up [27].

#### 3.6.5. Dietary Outcomes

The methods for assessing dietary outcomes and adherence varied among the studies (Table 2). One study employed daily food logs via a smartphone app [24], offering a selection of over 7000 foods. One paper utilised a food frequency questionnaire (FFQW65) [23], while another used 24-h recalls [27]. However, the most common method across studies was the use of food records [25,26,29], typically spanning 3 [25] to 7 [29] days.

Among the seven studies, five evaluated nutrient composition (Table 2) [24,25,26,27,29]. Of these, only one study investigated data at the food group level between groups [25]. Additionally, one study reported the top 10 most logged foods by participants based on whether they received the intervention or control intervention but did not report or quantify differences in intake between the groups [24]. Another study solely reported on the absolute value of the proportion of over/under intake fraction for estimated total energy [23]. 

## 4. Discussion

This review summarises the current evidence on the effectiveness of PPN and MNT in improving various outcomes related to glycaemic control, anthropometry, blood lipids, blood pressure, and diet among adults with prediabetes or MetS. Comparing interventions to standard care or non-personalised approaches showed evidence supporting PPN and MNT in improving certain glycaemic response outcomes like HbA1c, post-prandial glucose, and waist circumference, where the majority of studies (at least 75%) investigating these outcomes reported a significant between-group difference favouring the intervention (Figure 2, Table S3). However, mixed results were found for other outcomes such as fasting BGL, HOMA-IR, fasting insulin, BMI, weight, blood pressure, and blood lipids (Figure 2 and Table S3). Positive findings were identified for certain outcomes, such as mean CGM glucose, but were measured in only two studies. Variations in study design, including the types of PPN interventions and comparison groups utilised, as well as the frequency and duration of interventions, appeared to influence the magnitude of the reported changes.

The findings suggest that more intense and longer interventions seemed to have a greater positive effect, especially on outcomes like HbA1c and waist circumference. For instance, interventions with eight or more sessions or lasting 6 months or longer showed significant differences in HbA1c levels [24,27,29] and waist circumference [25,26,27] compared to less intensive and shorter studies. These results are not surprising, as HbA1c is a long-term marker of blood sugar levels, and longer interventions are expected to have a more notable impact on HbA1c results. Previous research has demonstrated that multiple encounters of MNT interventions are necessary to achieve desired outcomes in individuals with diabetes, such as HbA1c levels [31]. Similar findings have been observed for waist circumference, with longer interventions and more frequent sessions leading to greater weight loss [25,26,27]. These results are consistent with previous studies indicating that more than 28 sessions resulted in significantly better improvements in weight, BMI, waist circumference, HbA1c, and fasting blood glucose levels compared to those who received fewer sessions [32]. Given that obesity is a major risk factor for prediabetes and weight loss can reduce the risk of developing type 2 diabetes, personalised interventions and MNT may be effective in managing prediabetes and preventing its progression. However, more longer-term studies are necessary to better understand the relationship between the dose of intervention and its response in treating prediabetes.

Studies that compared mean CGM glucose levels [24,27] showed a significant decrease in favour of the PPN intervention groups. However, for other outcomes such as HOMA-IR, fasting insulin, fasting BGL, weight, BMI, total cholesterol, and blood pressure, significant differences favouring the intervention were reported in some studies, but the results were more mixed, as not all studies reported significant results. These findings are similar to a systematic review focusing on MNT and prediabetes, which found that MNT compared to standard care significantly improved HbA1c, fasting BGL, anthropometric measures, cholesterol levels, and blood pressure [33]. A study using MNT provided by dietitians reported a significant decrease within the intervention group in fasting BGL, weight, and HbA1c [28]. Differences in comparison groups and the level of precision and personalisation in the intervention were major sources of heterogeneity and may explain the inconsistencies across the studies in this review. As expected, studies that used standard care or generic information as the comparator tended to report a greater magnitude of difference in favour of the intervention group [23,25,26,27,29]. This is in contrast to studies where comparators, involving some level of guidance from a dietitian or personalisation, reported smaller differences between the control and intervention groups [24,28]. For instance, comparison groups that received small group sessions facilitated by a dietitian [28], or a personalised Mediterranean diet but did not receive the same level of precision and personalisation relating to the microbiome and other clinical or biological markers as the intervention group [24]. Additionally, the purpose of the intervention seemed to impact the outcomes differently. For example, a low-carbohydrate diet [27] successfully produced significant improvements in glycaemic and anthropometric-related measures relative to the comparison group but not for other clinical measures, such as blood pressure and blood lipids. This was in contrast to a study that investigated a tailored Mediterranean diet [25] provided by a nutritionist, which produced significant improvements in all glycaemic, anthropometric, and clinical measures and is likely explained by the manipulation of multiple dietary components in the diet, resulting in a more widespread effect.

### 4.1. Strengths and Limitations 

A major strength of the current review is the inclusion of only RCTs, which are the highest-ranked study designs in terms of evidence hierarchy. To minimise confounding effects on the results, studies were excluded if participants were also given medications, supplements, and physical activity as part of the intervention, thereby focusing solely on the effectiveness of dietary interventions. Another strength is that six out of the seven studies had positive ratings regarding quality assessment [23,24,25,26,27,28], therefore having a lower risk of bias. The limited evidence base and considerable variation in study design, interventions, comparison groups, and sample populations pose a challenge for drawing definitive conclusions and generalising results. For example, only one included paper personalised intake on factors other than diet and health data [24]. 

### 4.2. Recommendations

Further research is needed to determine the effectiveness of PPN among diverse population groups, especially since only two studies have investigated PPN interventions in individuals with MetS [25,26]. Moreover, different approaches to precision and personalisation need to be further explored. For example, only one study integrated advanced precision data, such as clinical and gut microbiome information, to predict individual post-prandial glycaemic responses to meals and further tailor their dietary intervention [24]. Advances in multi-omic technologies, including genomics and metabolomics, coupled with sophisticated data analysis techniques, have improved our understanding of individual variability and led to the identification of novel disease subgroups (subphenotypes) that impact clinical practice and disease understanding [2,34]. However, this review highlights the need for exploration and validation in the use of this information to guide PPN for people with pre-diabetes and MetS. At the same time, behavioural, psychological, and sociocultural factors are essential components of dietary prescription, which are core to MNT and key determinants of patient adherence and should not be underestimated in future PPN interventions [35]. Future studies should aim for larger sample sizes to enhance study power and detect statistically significant between-group differences more effectively. This will improve the generalisability of findings to a broader population. Finally, a major challenge identified during the paper selection process was lack of a standardised definition for PPN interventions. Establishing a universal definition for PPN will promote consistency and comparability across research within this field.

## 5. Conclusions

The current systematic review suggests that the use of PPN and MNT for managing prediabetes and MetS is promising, especially for interventions of longer duration and that offer more frequent sessions or contact with interventionists. More consistent improvements favouring PPN and MNT interventions were reported across studies examining outcomes such as HbA1c, post-prandial glucose, and waist circumference. However, further research is essential to enhance our understanding of PPN as a treatment for prediabetes and MetS. Different approaches to precision and personalisation should be further explored, for example, through combining MNT with contemporary factors such as genetic, epigenetic, metabolomic, and metagenomic data.

## Figures and Tables

**Table 1:** PICOS criteria for inclusion of final studies in this systematic review.

Category	Inclusion Criteria	Exclusion Criteria
Population	Adults (≥18 yrs) diagnosed with prediabetes or metabolic syndrome	Study participants diagnosed with a chronic disease (e.g., type 1 or 2 diabetes, cardiovascular disease, chronic kidney disease, or gestational diabetes)
Intervention	MNT (provided by a registered or accredited practising dietitian) or PPN	If the intervention included medications, surgeries, supplements, physical activity, or another lifestyle component, where the impact of the PPN intervention could not be isolated
Comparison	Standard care, habitual diet, or non-personalised dietary intervention	If the comparator or control was anything other than standard care or non-personalised/individualised dietary intervention
Outcome	Measures of glycaemic control [HbA1c, fasting blood glucose levels, post-prandial glucose/OGTT, Homeostatic Model Assessment for Insulin Resistance (HOMA-IR), insulin levels, and insulin sensitivity], anthropometry [weight, waist circumference, body mass index (BMI)], blood lipids, blood pressure, and reporting of dietary outcomes (nutrients, food groups, dietary patterns, diet quality)	Did not measure any outcome of interest relating to glycaemic control
Study design	Randomised control trials (RCTs) published after the year 2000	Studies that were not an RCT; studies not published in English

**Table 2:** Study characteristics and results summary of studies included in the systematic review examining the effect of PPN and MNT on adults with prediabetes.

First Author, Year, Country	Primary Risk	Participant Characteristics	RCT Design and Study Duration	Outcomes Measured	Intervention Prescribed By	Intervention Group(s) Conditions	Comparison Group(s) Conditions	Glycaemic Control Outcomes	Anthropometry Outcomes	Blood Lipids	Blood Pressure	Diet
Ben-Yacov et al., 2021, Israel [24]	Prediabetes	N = 225 (35–70 yrs; 41% male)	Parallel, 1 year	Glycaemic control (FBGL, 2-h and 5-h PPG (CGM), mean CGM glucose, HbA1c, HOMA-IR, insulin, and fructosamine), anthropometry (weight, BMI, waist circumference [WC]), blood lipids, blood pressure (SBP, DBP), and diet (daily food log via smartphone app)	*Intervention*: one-on-one counselling with a dietitian *Control*: one-on-one counselling with a dietitian	A tailored diet was created based on the participant’s personal predicted glucose responses using an algorithm that integrated clinical and gut microbiome features. Monthly in-person meetings with a dietitian during 6 months of follow-up and interim contact via telephone or email with a dietitian as needed. Diet recommendations were administered as menus	Monthly in-person meetings with a dietitian during 6 months of follow-up and interim contact via telephone or email with a dietitian as needed. The participants were encouraged to consume a Mediterranean diet consisting of whole foods and discouraged from consuming processed foods. Diet recommendations were administered as menus	Greater improvements in CGM mean above 140 (95% CI −1.29 to −0.66 h/day, *p* < 0.001), HbA1c (−0.14 to −0.02% [−1.5 to −0.2 mmol/mol], *p* = 0.007), 5-h PPG excursions (95% CI −12.3 to −7.6 mg/dL × h, *p* < 0.001), and mean CGM glucose (95% CI −7.0 to −3.22 mg/dL [−0.39 to −0.18 mmol/L], *p* < 0.001) in intervention group compared to control at 6 months. No significant difference between groups for fasting BGL, insulin, HOMA-IR, and 2-h PG (OGTT)	No significant difference between groups for BMI, weight, and fat (%) at 6 months	Greater improvements in triglycerides (95% CI −0.36 to −0.07 mmol/L [−31.51 to −6.11 mg/dL], *p* < 0.003) and HDL (95% CI 0.02–0.13 mmol/L [0.77–4.9 mg/dL], *p* = 0.003) in intervention group compared to control at 6 months. No significant difference for LDL and total cholesterol	No significant difference between groups	Significant difference between groups, with lower carbohydrate intake (−93.2, 95% CI −101.9 g to −84.4 g/d, *p* < 0.001), fibre intake (−10.8, 95% CI −12.9 to −8.7 g/day, *p* < 0.01), greater protein intake (+5.1, 95% CI +0.5 to +10.8 g/d, *p* < 0.001), and fat intake (+37.1, 95% CI +32.1 to +42.1 g/d, *p* < 0.001) and saturated fat intake (+11.5, 95% CI +9.7 to +13.2 g/d, *p* < 0.001) observed in the intervention group compared to the control group at 6 months. No significant difference between groups for energy intake. Reported participants’ top 10 most popular logged foods for the intervention and control diets, which differed; however, the authors did not report statistical between-group differences.
Cole et al., 2013, USA [28]	Prediabetes	N = 65 (≥18 yrs; 54% male)	Parallel, 1 year	Glycaemic control (FBGL, HbA1c), anthropometry (weight, BMI), blood lipids, and blood pressure (SBP, DBP)	*Intervention*: one-on-one counselling session with a dietitian *Control*: small medical appointments (SMA) with a dietitian, diabetes educator, and behaviour specialist or nurse	Participants attended at least one 45–60-min individualised counselling session with a dietitian following an initial 3-h prediabetes class. The dietitian discussed patients’ clinical outcomes and progress made in achieving lifestyle medication since the initial class, assisted in the development of SMART goals, and scheduled follow-up appointments if the patient desired	Participants participated in 3 90-min SMA that accommodated 6–8 participants and were supported by dietitians, diabetes educators, and behaviour specialists or nurses. Each participant also received 10 min of individual time to discuss clinical and biochemical measures, challenges, and smart goals	No significant difference between groups for FBGL and HbA1c	No significant difference between groups for weight and BMI	No significant difference between groups for total cholesterol, HDL, LDL, and triglycerides	No significant difference between groups for systolic and diastolic BP	n/a
Dorans et al., 2022, USA [27]	HbA1c 6.0–6.9%	N = 150 (40–70 yrs; 28% male)	Parallel, 6 months	Glycaemic control (FBGL, mean 24-h CGM glucose, HbA1c, HOMA-IR, and fasting insulin), anthropometry (weight, waist circumference [WC]), blood lipids, blood pressure (SBP, DBP), and diet (24-h recall)	*Intervention*: one-on-one counselling from an interventionist *Control*: received written information from the interventionist	Phase 1: The participant received behavioural counselling and key supplemental food with a carbohydrate target of less than 40 g. This phase involved weekly individual sessions for 4 weeks, followed by 4 small group sessions every other week and 4 telephone follow-ups. Phase 2: net carbohydrate target was less than 60 g. During this phase, participant attended three monthly group sessions and three telephone follow-ups	Participants received written information with standard dietary advice and did not receive ongoing recommendations. Participants were offered optional monthly educational sessions on topics unrelated to diet	Greater improvements in HbA1c (−0.23, 95% CI −0.32 to −0.14%, <0.001), FBGL (−10.3, 95% CI −15.6 to −4.9 mg/dL, *p* = 0.001), fasting insulin (−6.2, 95% CI −10.5 to −2 µIU/mL, *p* = 0.004), HOMA-IR (−2.4, 95% CI −3.7 to −1.1, *p* < 0.001) and mean 24-h CGM glucose (−7, 95% CI −13.8 to −0.1 mg/dL, *p* < 0.05) in intervention group compared to control at 6 months	Greater improvements in weight (−5.9, 95% CI −7.4 to −4.4 kg, *p* < 0.001) and waist circumference (−4.7, 95% CI −6.7 to −2.6 cm, *p* < 0.001) in intervention group compared to control at 6 months	No significant difference in HDL, LDL, and total-to-HDL between groups	No significant difference between groups for systolic and diastolic blood pressure	Did not report on difference between groups
Esposito et al., 2004, Italy [25]	Metabolic syndrome	N = 180 (≥18 yrs; 55% male)	Parallel, 2 years	Glycaemic control (BGL, HOMA-IR, fasting insulin), anthropometry (weight, BMI, waist circumference [WC]), blood lipids, blood pressure (SBP, DBP), and diet (3-day food record)	*Intervention*: one-on-one counselling provided by nutritionist *Control*: One-on-one visits with study personnel	Patients were given detailed advice through monthly group sessions. Received education on reducing dietary calories, goal-setting, and self-monitoring using food diaries. Behavioural and psychological counselling was also offered. The dietary advice was tailored to each patient on the basis of 3-day food records. The recommended composition of the dietary regimen was as follows: carbohydrates, 50% to 60%; proteins, 15% to 20%; total fat, less than 30%; saturated fat, less than 10%; and cholesterol consumption, less than 300 mg per day. Patients were advised to increase intake of fruit, vegetables, whole grains, walnuts, and olive oil. Received guidance on increasing their level of physical activity	Patients were given general oral and written information about health food choices at visits but were offered no specific individualized program. The general recommendation for macronutrient composition of the diet was carbohydrates, 50–60%; proteins, 15–20%; and total fat, <30%. Received guidance on increasing their level of physical activity	Greater improvements in BGL (−6, 95% CI −11 to −2 mg/dL. *p* < 0.001), insulin (−3.5, 95% CI −6.1 to −1.7 µIU/mL, *p* = 0.01), and HOMA score (−1.1, 95% CI −1.9 to −0.3, *p* < 0.001) in intervention group compared to control at 2 years	Greater improvements in weight (−2.8, 95% CI −5.1 to −0.5 kg, *p* < 0.001), BMI (−0.8, 95% CI −1.4 to −0.2 kg/m^2^, *p* = 0.01), and waist circumference (−2, 95% CI −3.5 to −0.5 cm, *p* = 0.01) in intervention group compared to control at 2 years	Greater improvements in total cholesterol (−9, 95% CI −17 to −1 mg/dL, *p* = 0.02), HDL (+3, 95% CI 0.8 to 5.2 mg/dL, *p* = 0.03), and triglycerides (−19, 95% CI −32 to −6 mg/dL, *p* = 0.001) in intervention group compared to control at 2 years	Greater improvement in systolic blood pressure (−3, 95% CI −5 to −1 mmHg, *p* = 0.01) and diastolic blood pressure (−2, 95% CI −3.5 to −0.5 mmHg, *p* = 0.03) in intervention group compared to control at 2 years	Greater reductions in energy intakes (−100, 95% CI −178 to −21 kcal/d, *p* < 0.001), fat (−1.4, 95% CI −2.8 to −0.2%, *p* = 0.02), SFA (−5.3, 95% CI −9.5 to −2.0%, *p* < 0.001), omega 6/3 ratio (−4.3, 95% CI −8.3 to −1, *p* < 0.001), and dietary cholesterol (−80, 95% CI −135 to −25 mg/d, *p* < 0.001) in intervention group compared to control. Greater increases in carbohydrate intake (+0.6, 95% CI +0.1 to +1.1%, *p* = 0.02), complex carbohydrate (+7, 95% CI +4 to +12%, *p* < 0.001), fibre (+16, 95% CI +4 to +30 g/day, *p* < 0.001), MUFA (+3, 95% CI +1.0 to +5.0%, *p* < 0.001), PUFA (+0.9, 95% CI +0.3 to +1.5, *p* = 0.01), and Omega 3 FA (+0.86, +0.25 to +1.4 g/day, *p* < 0.001) in intervention group compared to control at 2 years. No significant differences between groups for protein intake. At the food group level, significant improvement in olive oil (+8.2, 95% CI +3.3 to +12.4 g/d, *p* < 0.001), fruits, vegetables, nuts and legumes (+274, 95% CI +176 to +372 g/d, *p* < 0.001), and wholegrains (+103, 95% CI +45 to +159 g/d, *p* < 0.001) reported in the intervention group compared to the control group at 2 years. No significant between-group differences for alcohol consumption
Kolehmainen et al., 2007, Finland [26]	Impaired fasting glycemia or impaired glucose tolerance and features of metabolic syndrome	N = 46 (40–70 yrs; 43% male)	Parallel, 33 weeks	Glycaemic control (FBGL, fasting insulin, insulin sensitivity index, acute insulin response, glucose effectiveness index), anthropometry (weight, BMI, waist circumference [WC]), and diet (4-day food record)	*Intervention*: One-on-one counselling provided by a nutritionist *Control*: instructions by research personnel	Subjects underwent 12-week intensive weight reduction program followed by ~20-week weight maintenance. Received individual counselling from nutritionist based on food records, aiming to decrease energy intake. Follow-up meeting with nutritionist to check food record and discuss any difficulties. Subjects asked to maintain physical activity levels	Subjects were advised to continue normal lifestyle and to keep diet and exercise habits unchanged	No significant difference between groups for fasting FBGL, fasting insulin, insulin sensitivity index, acute insulin response, and glucose effectiveness index	Greater improvements in weight (*p* = 0.0002), BMI (*p* = 0.0002), and waist circumference (*p* = 0.0001) in intervention group compared to control at 33 weeks. Paper did not report mean difference/change between groups but reported mean change within groups from baseline	n/a	n/a	Significant decrease in MUFA intake (% energy) in the intervention versus the control (*p* = 0.013). No significant between-group difference for energy, protein, fat, SFA, PUFAs, carbohydrates, dietary cholesterol, fibre, and calcium intake
Pimentel et al., 2010, Brazil [29]	Impaired glucose tolerance and one other risk factor for T2DM	N = 51 (≥18 yrs 33–38% male at baseline)	Parallel, 1 year	Glycaemic control (FBGL, PPG and post-prandial insulin, HbA1c, HOMA-IR, fasting insulin), anthropometry (weight, BMI), blood lipids, and diet (7-day food record)	*Intervention*: one-on-one counselling and group counselling from nutritionist *Control*: no information provided	Participants received individual and group counselling from team of nutritionists. Consisted of discussion-format group sessions twice a month and individual sessions one per month. Instructions to improve diet quality provided orally and in written form	Control: No information provided.	Greater improvements in HbA1c (*p* < 0.05), PPG (*p* < 0.05), post-prandial insulin (*p* < 0.05), and FBGL (*p* < 0.05) in the intervention group compared to control at 1 year. No significant difference between groups for fasting insulin and HOMA-IR. Paper did not report mean difference/change between groups but reported significance	No significant difference between groups for weight and BMI	Greater improvements in total cholesterol (*p* < 0.05) in intervention group compared to control at 1 year. No significant difference reported between groups for HDL, LDL, and triglycerides Paper did not report mean difference/change between groups but reported significance	n/a	Greater improvements in dietetic (dietary) cholesterol (*p* < 0.05) in intervention group compared to control at 1 year. No significant difference between groups for energy, carbohydrate, protein, fat, and saturated fat. Paper did not report mean difference/change between groups but reported significance
Watanabe et al., 2003, Japan [23]	Borderline diabetes (patients with 1-h PG ≥10 mmol/L)	N = 156 (35–70 yrs; 100% male)	Parallel, 1 year	Glycaemic control (FBGL, 1-h, and 2-h PPG) and diet (FFQW65)	*Intervention*: one-on-one counselling was provided by a nutritionist, and additional resources were sent via post *Control*: Provided with oral and written information by the interventionist	Phase 1: Individualised counselling was received using a booklet explaining the concepts of the new dietary education (NDE) program. Information was provided on dietary intake based on a food frequency questionnaire and motivation to improve dietary practices. Phase 2: The following resources were sent via post: letter encouraging the subject to improve dietary habits, examples of menus corresponding to the subject’s RDA and information to confirm the necessity of blood glucose control	General oral and written information about results of health examination and food frequency questionnaire without detailed explanation. Received conventional group counselling using leaflet with general information on prevention of lifestyle-related diseases	Greater improvement in 2-h PPG (−15.2%, 95% CI −22.0 to −8.4%, *p* < 0.001) in the intervention group compared to control at 1 year. No significant difference for fasting BGL and 1-h PPG between groups	n/a Measured at baseline but not reported at follow-up visit	n/a Measured at baseline but not reported at follow-up visit	n/a Measured at baseline but not reported at follow-up visit	Greater improvement in daily absolute value of “overintake/underintake fraction” for total energy intake (%) (−6.0%, 95% CI −9.8 to −2.2%, *p* = 0.002) and dinner (−15.3%, 95% CI −24.6 to −6.0%, *p* = 0.002) in the intervention group compared to control at 1 year. No significant difference for absolute value of “overintake/underintake fraction” for total energy intake (%) during breakfast and lunch between groups

BGL (Blood Glucose Level), BMI (Body Mass Index), BP (Blood Pressure), FBGL (Fasting Blood Glucose Level), HbA1c (Glycated Haemoglobin), HDL (High-Density Lipoprotein), HOMA-IR (Homeostatic Model Assessment for Insulin Resistance), LDL (Low-Density Lipoprotein), MUFA (Mono-Unsaturated Fatty Acid), N/A (Not Available), PUFA (Poly-Unsaturated Fatty Acid), SFA (Saturated Fatty Acid), Yrs (Years), PPG (Post-Prandial Glucose).

## Data Availability

Given that this was a systematic review, all data used for this study are already published and publicly available.

## Supplementary Materials

The following supporting information can be downloaded at: https://www.mdpi.com/article/10.3390/nu16101479/s1, Table S1: PRISMA Reporting Guidelines Checklist; Table S2: Academy of Nutrition and Dietetic Quality criteria checklist for included studies in the systematic review examining the effect of PN and MNT for adults with prediabetes; Table S3: Summary of between-group differences between the intervention and comparison groups for outcomes of interest (Source data for Figure 2 in the manuscript). Tables S4–S9: Search string for each database.
