# Supplementary material for: Do Precision and Personalised Nutrition Interventions Improve Risk Factors in Adults with Prediabetes or Metabolic Syndrome? A Systematic Review of Randomised Controlled Trials
Source: Nutrients. 2024 May 14;16(10):1479. doi: 10.3390/nu16101479 (PMC11124316; doi:10.3390/nu16101479)
Supplement: Supplementary file 1 [file nutrients-16-01479-s001.zip › nutrients-2962180-supplementary.pdf]

Supplementary Materials

Title: Do precision and personalised nutrition interventions improve risk factors in adults with prediabetes or metabolic syndrome? A systematic review of randomised controlled trials

TABLE OF CONTENTS

**Table S1**	PRISMA Reporting Guidelines Checklist	Pages 1-4
**Table S2**	Academy of Nutrition and Dietetic Quality criteria checklist for included studies in the systematic review examining the effect of PN and MNT for adults with prediabetes.	Pages 5-8
**Table S3**	Summary of between-group differences between the intervention and comparison groups for outcomes of interest (Source data for Figure 2 in the manuscript).	Page 9-10
**Table S4- S9**	Search string for each database	Pages 11-19

Supplementary Table S1 PRISMA Reporting Guidelines Checklist

Section and Topic	Item #	Checklist item	Location where item is reported
**TITLE**			
Title	1	Identify the report as a systematic review.	Page 1
**ABSTRACT**			
Abstract	2	See the PRISMA 2020 for Abstracts checklist.	Page 1
**INTRODUCTION**			
Rationale	3	Describe the rationale for the review in the context of existing knowledge.	page 2
Objectives	4	Provide an explicit statement of the objective(s) or question(s) the review addresses.	Page 2
**METHODS**			
Eligibility criteria	5	Specify the inclusion and exclusion criteria for the review and how studies were grouped for the syntheses.	Page 3
Information sources	6	Specify all databases, registers, websites, organisations, reference lists and other sources searched or consulted to identify studies. Specify the date when each source was last searched or consulted.	Page 3
Search strategy	7	Present the full search strategies for all databases, registers and websites, including any filters and limits used.	Page 3 & Figures S1-S6
Selection process	8	Specify the methods used to decide whether a study met the inclusion criteria of the review, including how many reviewers screened each record and each report retrieved, whether they worked independently, and if applicable, details of automation tools used in the process.	Page 3-4
Data collection process	9	Specify the methods used to collect data from reports, including how many reviewers collected data from each report, whether they worked independently, any processes for obtaining or confirming data from study investigators, and if applicable, details of automation tools used in the process.	Page 3-4
Data items	10a	List and define all outcomes for which data were sought. Specify whether all results that were compatible with each outcome domain in each study were sought (e.g. for all measures, time points, analyses), and if not, the methods used to decide which results to collect.	Page 4
	10b	List and define all other variables for which data were sought (e.g. participant and intervention characteristics, funding sources). Describe any assumptions made about any missing or unclear information.	Page 4
Study risk of bias assessment	11	Specify the methods used to assess risk of bias in the included studies, including details of the tool(s) used, how many reviewers assessed each study and whether they worked independently, and if applicable, details of automation tools used in the process.	Page 4 & Table S2

Effect measures	12	Specify for each outcome the effect measure(s) (e.g. risk ratio, mean difference) used in the synthesis or presentation of results.	Page 3
Synthesis methods	13a	Describe the processes used to decide which studies were eligible for each synthesis (e.g. tabulating the study intervention characteristics and comparing against the planned groups for each synthesis (item #5)).	Page 4
	13b	Describe any methods required to prepare the data for presentation or synthesis, such as handling of missing summary statistics, or data conversions.	Page 4
	13c	Describe any methods used to tabulate or visually display results of individual studies and syntheses.	Page 4
	13d	Describe any methods used to synthesize results and provide a rationale for the choice(s). If meta-analysis was performed, describe the model(s), method(s) to identify the presence and extent of statistical heterogeneity, and software package(s) used.	Page 4
	13e	Describe any methods used to explore possible causes of heterogeneity among study results (e.g. subgroup analysis, meta-regression).	Page 4
	13f	Describe any sensitivity analyses conducted to assess robustness of the synthesized results.	NA
Reporting bias assessment	14	Describe any methods used to assess risk of bias due to missing results in a synthesis (arising from reporting biases).	Page 4
Certainty assessment	15	Describe any methods used to assess certainty (or confidence) in the body of evidence for an outcome.	NA
**RESULTS**			
Study selection	16a	Describe the results of the search and selection process, from the number of records identified in the search to the number of studies included in the review, ideally using a flow diagram.	Page 15
	16b	Cite studies that might appear to meet the inclusion criteria, but which were excluded, and explain why they were excluded.	Page 15
Study characteristics	17	Cite each included study and present its characteristics.	Page 15
Risk of bias in studies	18	Present assessments of risk of bias for each included study.	Page 16 & Table S2
Results of individual studies	19	For all outcomes, present, for each study: (a) summary statistics for each group (where appropriate) and (b) an effect estimate and its precision (e.g. confidence/credible interval), ideally using structured tables or plots.	Page 6-14
Results of syntheses	20a	For each synthesis, briefly summarise the characteristics and risk of bias among contributing studies.	Page 6-14
	20b	Present results of all statistical syntheses conducted. If meta-analysis was done, present for each the summary estimate and its precision (e.g. confidence/credible interval) and measures of statistical heterogeneity. If comparing groups, describe the direction of the effect.	NA
	20c	Present results of all investigations of possible causes of heterogeneity among study results.	Page 16-17
	20d	Present results of all sensitivity analyses conducted to assess the robustness of the synthesized results.	NA
Reporting biases	21	Present assessments of risk of bias due to missing results (arising from reporting biases) for each synthesis assessed.	Page 16 & Table S2

Certainty of evidence	22	Present assessments of certainty (or confidence) in the body of evidence for each outcome assessed.	NA
**DISCUSSION**			
Discussion	23a	Provide a general interpretation of the results in the context of other evidence.	Page 19-20
	23b	Discuss any limitations of the evidence included in the review.	Page 20
	23c	Discuss any limitations of the review processes used.	Page 20
	23d	Discuss implications of the results for practice, policy, and future research.	Page 20-21
**OTHER INFORMATION**			
Registration and protocol	24a	Provide registration information for the review, including register name and registration number, or state that the review was not registered.	Page 2
	24b	Indicate where the review protocol can be accessed, or state that a protocol was not prepared.	Page 2
	24c	Describe and explain any amendments to information provided at registration or in the protocol.	NA
Support	25	Describe sources of financial or non-financial support for the review, and the role of the funders or sponsors in the review.	Title page
Competing interests	26	Declare any competing interests of review authors.	Title page
Availability of data, code and other materials	27	Report which of the following are publicly available and where they can be found: template data collection forms; data extracted from included studies; data used for all analyses; analytic code; any other materials used in the review.	NA

From: Page MJ, McKenzie JE, Bossuyt PM, Boutron I, Hoffmann TC, Mulrow CD, et al. The PRISMA 2020 statement: an updated guideline for reporting systematic reviews. BMJ 2021;372:n71. doi: 10.1136/bmj.n71

For more information, visit: http://www.prisma-statement.org/

Table S2. Academy of Nutrition and Dietetic Quality criteria checklist for included studies in the systematic review examining the effect of PN and MNT for adults with prediabetes.

Study reference	Relevance questions				Validity questions										Overall rating
	Q1	Q2	Q3	Q4	Q1	Q2	Q3	Q4	Q5	Q6	Q7	Q8	Q9	Q10	
Watanabe et al. 2003	Yes	Yes	Yes	Yes	Yes	Yes	Yes	Yes	Yes	Yes	Yes	Yes	Yes	Yes	+
Ben-Yacov et al. 2021	Yes	Yes	Yes	Yes	Yes	Yes	Yes	Yes	Yes	Yes	Yes	Yes	Yes	?	+
Esposito et al. 2004	Yes	Yes	Yes	Yes	Yes	Yes	Yes	Yes	Yes	Yes	Yes	Yes	Yes	Yes	+
Kolehmainen et al 2007	Yes	Yes	Yes	Yes	Yes	Yes	Yes	?	No	Yes	Yes	Yes	Yes	Yes	+
Doran et al. 2022	Yes	Yes	Yes	Yes	Yes	Yes	Yes	Yes	Yes	Yes	Yes	Yes	Yes	Yes	+
Cole et al. 2013	Yes	Yes	Yes	Yes	Yes	Yes	Yes	Yes	No	Yes	Yes	Yes	Yes	Yes	+
Pimental et al. 2010	Yes	Yes	Yes	Yes	Yes	Yes	?	Yes	No	?	Yes	Yes	Yes	?	Ø

?: Unclear. +: Positive. Ө: Neutral. -: Negative. n/a: not applicable.

Quality Criteria Checklist Questions

Relevance

1. **Would implementing the studied intervention or procedure (if found successful) result in improved outcomes for the patients/clients/population group? (NA for some epidemiological studies)**
2. **Did the authors study an outcome (dependent variable) or topic the patients/clients/population group would care about?**
3. **Is the focus of the intervention or procedure (independent variable) or topic of study a common issue of concern to dietetics practice?**
4. **Is the intervention or procedure feasible? (NA for some epidemiological studies)**

Validity

1. **Was the research question clearly stated?**

1.1 Was the specific intervention(s) or procedure (independent variable(s)) identified?
1.2 Was the outcome(s) (dependent variable(s)) clearly indicated?
1.3 Were the target population and setting specified?

2. Was the selection of study subjects/patients free from bias?

2.1 Were inclusion/exclusion criteria specified (e.g., risk, point in disease progression, diagnostic or prognosis criteria), and with sufficient detail and without omitting criteria critical to the study?

2.2 Were criteria applied equally to all study groups?

2.3 Were health, demographics, and other characteristics of subjects described?

2.4 Were the subjects/patients a representative sample of the relevant population?

3. Were study groups comparable?

3.1 Was the method of assigning subjects/patients to groups described and unbiased? (Method of randomization identified if RCT)

3.2 Were distribution of disease status, prognostic factors, and other factors (e.g., demographics) similar across study groups at baseline?

3.3 Were concurrent controls used? (Concurrent preferred over historical controls.)

3.4 If cohort study or cross-sectional study, were groups comparable on important confounding factors and/or were pre-existing differences accounted for by using appropriate adjustments in statistical analysis?

3.5 If case control study, were potential confounding factors comparable for cases and controls? (If case series or trial with subjects serving as own control, this criterion is not applicable. Criterion may not be applicable in some cross-sectional studies.)

3.6 If diagnostic test, was there an independent blind comparison with an appropriate reference standard (e.g., "gold standard")?

4. Was method of handling withdrawals described?

4.1 Were follow up methods described and the same for all groups?

4.2 Was the number, characteristics of withdrawals (i.e., dropouts, lost to follow up, attrition rate) and/or response rate (cross-sectional studies) described for each group? (Follow up goal for a strong study is 80%.)

4.3 Were all enrolled subjects/patients (in the original sample) accounted for?

4.4 Were reasons for withdrawals similar across groups?

4.5 If diagnostic test, was decision to perform reference test not dependent on results of test under study?

5. Was blinding used to prevent introduction of bias?

5.1 In intervention study, were subjects, clinicians/practitioners, and investigators blinded to treatment group, as appropriate?

5.2 Were data collectors blinded for outcomes assessment? (If outcome is measured using an objective test, such as a lab value, this criterion is assumed to be met.)

5.3 In cohort study or cross-sectional study, were measurements of outcomes and risk factors blinded?

5.4 In case control study, was case definition explicit and case ascertainment not influenced by exposure status?
5.5 In diagnostic study, were test results blinded to patient history and other test results?

6. Were intervention/therapeutic regimens/exposure factor or procedure and any comparison(s) described in detail? Were intervening factors described?

6.1 In RCT or other intervention trial, were protocols described for all regimens studied?
6.2 In observational study, were interventions, study settings, and clinicians/provider described?
6.3 Was the intensity and duration of the intervention or exposure factor sufficient to produce a meaningful effect?
6.4 Was the amount of exposure and, if relevant, subject/patient compliance measured?
6.5 Were co-interventions (e.g., ancillary treatments, other therapies) described?
6.6 Were extra or unplanned treatments described?
6.7 Was the information for 6.4, 6.5, and 6.6 assessed the same way for all groups?
6.8 In diagnostic study, were details of test administration and replication sufficient?

7. Were outcomes clearly defined and the measurements valid and reliable?

7.1 Were primary and secondary endpoints described and relevant to the question?
7.2 Were nutrition measures appropriate to question and outcomes of concern?
7.3 Was the period of follow-up long enough for important outcome(s) to occur?
7.4 Were the observations and measurements based on standard, valid, and reliable data collection instruments/tests/procedures?
7.5 Was the measurement of effect at an appropriate level of precision?
7.6 Were other factors accounted for (measured) that could affect outcomes?
7.7 Were the measurements conducted consistently across groups?

8. Was the statistical analysis appropriate for the study design and type of outcome indicators?

8.1 Were statistical analyses adequately described the results reported appropriately?
8.2 Were correct statistical tests used and assumptions of test not violated?
8.3 Were statistics reported with levels of significance and/or confidence intervals?
8.4 Was "intent to treat" analysis of outcomes done (and as appropriate, was there an analysis of outcomes for those maximally exposed or a dose-response analysis)?
8.5 Were adequate adjustments made for effects of confounding factors that might have affected the outcomes (e.g., multivariate analyses)?

8.6 Was clinical significance as well as statistical significance reported?
8.7 If negative findings, was a power calculation reported to address type 2 error?

9. Are conclusions supported by results with biases and limitations taken into consideration?
9.1 Is there a discussion of findings?
9.2 Are biases and study limitations identified and discussed?

10. Is bias due to study's funding or sponsorship unlikely?
10.1 Were sources of funding and investigators' affiliations described?
10.2 Was there no apparent conflict of interest

Table S3. Summary of between-group differences between the intervention and comparison groups for outcomes of interest (Source data for Figure 2 in the manuscript).

	**Glycaemic control**						**Anthropometry**			**Blood pressure**		**Blood lipids**			
Author, year	BGL	Post-prandial glucose	Mean glucose (CGM)	HbA1c	HOMA-IR	Fasting Insulin	Weight	Waist circumference	BMI	Systolic blood pressure	Diastolic blood pressure	Total cholesterol	LDL-cholesterol	HDL-cholesterol	Triglycerides
Ben-Yacov et al 2021, Israel[24]	NS	↓[¥] (5-hrs) NS (2-hrs)	↓	↓	NS	NS[§]	NS	NS	NS	NS	NS	NS	NS	↑	↓
Cole et al 2013, USA[28]	NS			NS			NS		NS	NS	NS	NS	NS	NS	NS
Doran et al 2022, USA[27]	↓		↓	↓	↓	↓	↓	↓		NS	NS	NS	NS	NS	
Esposito et al 2004, Italy[25]	↓*				↓	↓	↓	↓	↓	↓	↓	↓		↑	↓
Kolehmainen et al 2007, Finland[26]	NS					NS	↓	↓	↓	NS	NS				

Pimentel et al 2010, Brazil[29]	↓	↓#		↓	NS	NS	NS		NS			↓	NS	NS	NS
Watanabe et al 2003, Japan[23]	NS	↓ (2-hrs) NS (1-hr)													

The direction of the arrow indicates the statistical differences between groups: up signifies an increase in the intervention group compared to the control group, while down indicates a decrease in the intervention group compared to the control group. “NS” denotes not statistically significant, and blank boxes indicate that the study did not report or measure that outcome. *Only study that didn’t specify if blood/ plasma glucose levels were measured in a fasted state. # didn't specify timing when post-prandial glucose was measured. ¥ Post-prandial glucose was calculated from continuous glucose monitor (CGM) data. § Did not specify if insulin levels were measured in a fasted state. BGL (Blood Glucose Level).

Supplementary Tables. Database search strategy

Table S4. Search strategy for SCOPUS database.

(TITLE-ABS-KEY ("Prediabet*" OR "risk of diabet*" OR "Impaired glucose tolerance" OR "insulin resistance" OR "Prediabetic state" OR "Hyperglyc*" OR "pre-diabet*" OR "impaired fasting glucose" OR "impaired fasting glycaem*" OR "impaired fasting glycem*") AND TITLE-ABS-KEY ("personalised diet*" OR "personalized diet*" OR "nutrigenomic*" OR "individualised nutrition*" OR "individualized nutrition*" OR gene OR genes OR dna OR genomic* OR "genotype" OR "medical nutrition therap*" OR "diet therapy" OR "counsel*") AND TITLE-ABS-KEY (diet* OR nutrition*) AND TITLE-ABS-KEY (effect* OR outcome* OR change* OR impact* OR modif*) AND TITLE-ABS-KEY (recommendation* OR advice OR information OR program OR counsel* OR intervention*) AND TITLE-ABS-KEY ("randomised controlled trial" OR "RCT" OR "randomized controlled trial" OR "randomised intervention" OR "randomized intervention")) AND PUBYEAR > 1999 AND PUBYEAR > 1999

Table S5. Search strategy for Cochrane database.

ID	Search
#1	MeSH descriptor: [Prediabetic State] this term only
#2	(prediabet*):ti,ab,kw
#3	(risk of diabet*):ti,ab,kw
#4	(Impaired glucose tolerance):ti,ab,kw
#5	(insulin resistance):ti,ab,kw
#6	(Prediabetic state):ti,ab,kw
#7	MeSH descriptor: [Insulin Resistance] this term only
#8	MeSH descriptor: [Glucose Intolerance] this term only
#9	MeSH descriptor: [Hyperglycemia] this term only
#10	(Hyperglyc*):ti,ab,kw
#11	(pre-diabet*):ti,ab,kw
#12	(impaired fasting glucose):ti,ab,kw
#13	(impaired fasting glycaem*):ti,ab,kw
#14	(impaired fasting glycem*):ti,ab,kw
#15	1 OR 2 OR 3 OR 4 OR 5 OR 6 OR 7 OR 8 OR 9 OR 10 O11 OR 12 OR 13 OR 14
#16	(personalised diet*):ti,ab,kw
#17	(personalized diet*):ti,ab,kw
#18	MeSH descriptor: [Nutrigenomics] this term only
#19	(nutrigenomic*):ti,ab,kw
#20	(individualised nutrition*):ti,ab,kw
#21	(individualized nutrition*):ti,ab,kw
#22	MeSH descriptor: [Genes] this term only
#23	(gene):ti,ab,kw
#24	(genes):ti,ab,kw
#25	MeSH descriptor: [DNA] this term only
#26	(dna):ti,ab,kw
#27	MeSH descriptor: [Genomics] this term only
#28	(genomic*):ti,ab,kw
#29	MeSH descriptor: [Genotype] this term only
#30	(genotype):ti,ab,kw
#31	MeSH descriptor: [Nutrition Therapy] this term only
#32	(medical nutrition therap*):ti,ab,kw
#33	MeSH descriptor: [Diet Therapy] this term only
#34	(diet therapy):ti,ab,kw
#35	(counsel*):ti,ab,kw
#36	16 OR 17 OR 18 OR 19 OR 20 OR 21 OR 22 OR 23 OR 24 OR 25 OR 26 OR 27 OR 28 OR 29 OR 30 OR 31 OR 32 OR 33 OR 34 OR 35
#37	MeSH descriptor: [Diet] explode all trees
#38	(diet*):ti,ab,kw
#39	(nutrition*):ti,ab,kw
#40	37 OR 38 OR 39

#41	(effect*):ti,ab,kw
#42	(outcome*):ti,ab,kw
#43	(change*):ti,ab,kw
#44	(impact*):ti,ab,kw
#45	(modif*):ti,ab,kw
#46	41 OR 42 OR 43 OR 44 OR 45
#47	(recommendation*):ti,ab,kw
#48	(advice):ti,ab,kw
#49	(information):ti,ab,kw
#50	(program):ti,ab,kw
#51	(counsel*):ti,ab,kw
#52	(intervention*):ti,ab,kw
#53	47 OR 48 OR 49 OR 50 OR 51 OR 52
#54	(randomised controlled trial):ti,ab,kw
#55	(RCT):ti,ab,kw
#56	MeSH descriptor: [Randomized Controlled Trial] this term only
#57	(randomized controlled trial):ti,ab,kw
#58	(randomised intervention):ti,ab,kw
#59	(randomized intervention):ti,ab,kw
#60	54 OR 55 OR 56 OR 57 OR 58 OR 59
#61	15 AND 36 AND 40 AND 46 AND 53 AND 60 with Cochrane Library publication date Between Jan 2000 and Dec 2023

Table S6. Search strategy for CINAHL database.

Search no	Search term
S1	Prediabet*
S2	Risk of diabet*
S3	Impaired glucose tolerance
S4	insulin resistance
S5	Prediabetic state
S6	Hyperglyc*
S7	pre-diabet*
S8	impaired fasting glucose
S9	impaired fasting glycaem*
S10	impaired fasting glycem*
S11	S1 OR S2 OR 53 OR S4 OR SS OR S6 OR S7 OR S8 OR S9 OR S10
S12	personalised diet*
S13	personalized diet*
S14	nutrigenomic*
S15	individualised nutrition*
S16	individualized nutrition*
S17	gene
S18	genes
S19	dna
S20	genomic*
S21	genotype
S22	medical nutrition therap*
S23	diet therapy
S24	counsel*
S25	S12 OR S13 OR S14 OR S15 OR S16 OR S17 OR S18 OR S19 OR S20 OR S21 OR S22 OR S23 OR S24
S26	diet*
S27	nutrition*
S28	S26 OR S27
S29	effect*
S30	outcome*
S31	change*
S32	impact*
S33	modif*
S34	S29 OR S30 OR S31 OR S32 OR S33
S35	recommendation*
S36	advice
S37	information
S38	program

S39	counsel*
S40	intervention*
S41	S35 OR 536 OR S37 OR 538 OR S39 OR S40
S42	S11 AND S25 AND S28 AND S34 AND S41
S43	S11 AND S25 AND S28 AND S34 AND S41 (Limiters - Published Date: 20000101-20231231; Publication Type: Randomized Controlled Trial)

Table S7. Search strategy for Medline database.

Search no.	Search item
1	Prediabetic State/ or prediabet*.mp.
2	risk of diabet*.mp.
3	impaired glucose tolerance.mp. or Glucose Intolerance/
4	insulin resistance.mp. or Insulin Resistance/
5	Hyperglycemia/ or hyperglyc*.mp.
6	pre-diabet*.mp.
7	impaired fasting glucose.mp.
8	impaired fasting glycaem*.mp.
9	impaired fasting glycem*.mp.
10	1 or 2 or 3 or 4 or 5 or 6 or 7 or 8 or 9
11	personalised diet*.mp.
12	personalized diet*.mp.
13	Nutrigenomics/ or nutrigenomic*.mp.
14	Nutrition Therapy/ or individualised nutrition*.mp.
15	individualized nutrition*.mp.
16	gene.mp. or Genes/
17	genes.mp or Genes/
18	DNA/ or dna.mp.
19	genomic*.mp.
20	genotype.mp. or Genotype/
21	medical nutrition therap*.mp.
22	diet therapy.mp. or Diet Therapy/
23	counsel*.mp.
24	11 or 12 or 13 or 14 or 15 or 16 or 17 or 18 or 19 or 20 or 21 or 22 or 23
25	Diet/ or diet*.mp.
26	nutrition*.mp.
27	25 or 26
28	effect*.mp.
29	outcome*.mp.
30	change*.mp.
31	impact*.mp.
32	modif*.mp.
33	28 or 29 or 30 or 31 or 32
34	recommendation*.mp.
35	advice.mp.
36	information.mp.
37	program.mp.
38	counsel*.mp.
39	intervention*.mp.
40	34 or 35 or 36 or 37 or 38 or 39
41	10 and 24 and 27 and 33 and 40
42	limit 41 to (yr="2000-Current" and randomized controlled trial)

Table S8. Search strategy for PyscINFO database.

Search no.	Search Term
1	prediabet*.mp.
2	risk of diabet*.mp.
3	Impaired glucose tolerance.mp.
4	insulin resistance.mp.
5	Prediabetic state.mp.
6	Hyperglyc*.mp.
7	pre-diabet*.mp.
8	impaired fasting glucose.mp.
9	impaired fasting glycaem*.mp.
10	impaired fasting glycem*.mp.
11	1 or 2 or 3 or 4 or 5 or 6 or 7 or 8 or 9 or 10
12	Nutrition/ or Diets/ or personalised diet*.mp.
13	personalized diet*.mp.
14	Personalization/ or nutrigenomic*.mp.
15	individualised nutrition*.mp.
16	individualized nutrition*.mp.
17	Genes/ or gene.mp.
18	Genes/ or genes.mp.
19	dna.mp. or DNA/
20	genomic*.mp. or Genomics/
21	genotype.mp. or Genotypes/
22	medical nutrition therap*.mp.
23	diet therapy.mp.
24	counsel*.mp.
25	12 or 13 or 14 or 15 or 16 or 17 or 18 or 19 or 20 or 21 or 22 or 23 or 24
26	diet*.mp.
27	Diets/ or Nutrition/ or nutrition*.mp.
28	26 or 27
29	effect*.mp.
30	outcome*.mp.
31	change*.mp.
32	impact*.mp.
33	modif*.mp.
34	29 or 30 or 31 or 32 or 33
35	recommendation*.mp.
36	advice.mp
37	Information/or information.mp.
38	program.mp.
39	counsel*.mp.
40	intervention*.mp.
41	35 or 36 or 37 or 38 or 39 or 40
42	randomised controlled trial.mp.
43	RCT.mp.
44	Randomized Controlled Trials/ or randomized controlled trial.mp.
45	randomised intervention.mp.
46	randomized intervention.mp.
47	42 or 43 or 44 or 45 or 46

48	11 and 25 and 28 and 34 and 41 and 47
49	limit 48 to "rr="2000-202

Table S9. Search strategy for Embase database.

Search no.	Search item
1	Prediabetic State/ or impaired glucose tolerance/or Prediabet*.mp.
2	risk of diabet*.mp.
3	Impaired glucose tolerance.mp. or impaired glucose tolerance/
4	insulin resistance.mp. or insulin resistance/
5	Prediabetic state.mp.
6	hyperglycemia/ or Hyperglyc*.mp.
7	pre-diabet*.mp.
8	impaired fasting glucose.mp.
9	impaired fasting glycaem*.mp.
10	impaired fasting glycem*.mp.
11	1 or 2 or 3 or 4 or 5 or 6 or 7 or 8 or 9 or 10
12	nutrition/ or personalised diet*.mp. or diet/
13	personalized nutrition/ or personalized diet*.mp.
14	nutrigenomics/ or nutrigenomic*.mp.
15	individualised nutrition*.mp.
16	diet therapy/ or individualized nutrition*.mp.
17	gene.mp. or gene/
18	genes.mp. or gene/
19	dna.mp. or DNA/
20	genomic*.mp.
21	genotype/orgenotype*.mp.
22	medical nutrition therap*.mp.
23	diet therapy.mp. or diet therapy/
24	counsel*.mp.
25	12 or 13 or 14 or 15 or 16 or 17 or 18 or 19 or 20 or 21 or 22 or 23 or 24
26	diet/ or diet*.mp.
27	nutrition/ or personalized nutrition/or nutrition*.mp.
28	26 or 27
29	effect*.mp.
30	outcome*.mp.
31	change*.mp.
32	impact*.mp.
33	modif*.mp.
34	29 or 30 or 31 or 32 or 33
35	recommendation*.mp.
36	advice.mp
37	information/ or information.mp.
38	program.mp.
39	counsel*.mp.
40	intervention*.mp.
41	35 or 36 or 37 or 38 or 39 or 40
42	11 and 25 and 28 and 34 and 41
43	limit 42 to (randomized controlled trial and yr="2000-2023")
